# Oncogenic Serine 45-Deleted β-Catenin Remains Susceptible to Wnt Stimulation and APC Regulation in Human Colonocytes

**DOI:** 10.3390/cancers12082114

**Published:** 2020-07-30

**Authors:** Taybor W. Parker, Aaron J. Rudeen, Kristi L. Neufeld

**Affiliations:** Department of Molecular Biosciences, University of Kansas, Lawrence, KS 66049, USA; parkertw@ku.edu (T.W.P.); aaron.rudeen@ku.edu (A.J.R.)

**Keywords:** Wnt signaling, just-right signaling, APC, β-catenin, colorectal cancer

## Abstract

The Wnt/β-catenin signaling pathway is deregulated in nearly all colorectal cancers (CRCs), predominantly through mutation of the tumor suppressor *Adenomatous Polyposis Coli* (*APC*). *APC* mutation is thought to allow a “just-right” amount of Wnt pathway activation by fine-tuning β-catenin levels. While at a much lower frequency, mutations that result in a β-catenin that is compromised for degradation occur in a subset of human CRCs. Here, we investigate whether one such “stabilized” β-catenin responds to regulatory stimuli, thus allowing β-catenin levels conducive for tumor formation. We utilize cells harboring a single mutant allele encoding Ser45-deleted β-catenin (β-catΔS45) to test the effects of Wnt3a treatment or APC-depletion on β-catΔS45 regulation and activity. We find that APC and β-catΔS45 retain interaction with Wnt receptors. Unexpectedly, β-catΔS45 accumulates and activates TOPflash reporter upon Wnt treatment or APC-depletion, but only accumulates in the nucleus upon APC loss. Finally, we find that β-catenin phosphorylation at GSK-3β sites and proteasomal degradation continue to occur in the absence of Ser45. Our results expand the current understanding of Wnt/β-catenin signaling and provide an example of a β-catenin mutation that maintains some ability to respond to Wnt, a possible key to establishing β-catenin activity that is “just-right” for tumorigenesis.

## 1. Introduction

Discovered nearly 40 years ago, the Wnt signaling pathway has proven essential for many cellular functions, including proliferation, polarity, developmental cell-fate determination, and tissue homeostasis [1]. Consequently, the Wnt pathway is often deregulated in cancer and other diseases. Normal colon tissue homeostasis is dependent on well-controlled Wnt signaling, as Wnt pathway components are mutated in over 90% of colorectal cancers (CRCs) [2,3,4].

The key downstream effector molecule in the Wnt pathway is the transcription cofactor β-catenin. In the absence of a Wnt signal, a cytoplasmic β-catenin destruction complex efficiently catalyzes the proteasome-mediated degradation of β-catenin. The core components of the complex include two scaffolding proteins, Adenomatous Polyposis Coli (APC) and Axin, as well as two kinases, GSK-3β and CK1-α [5]. In the absence of ligands, the complex binds and phosphorylates β-catenin, leading to β-TrCP-mediated ubiquitination and proteasomal degradation [6,7,8]. Binding of Wnt to membrane-bound coreceptors Frizzled and LRP5/6 results in inhibition of the β-catenin destruction complex through an incompletely resolved mechanism, followed by β-catenin accumulation, nuclear translocation, and activation of the Wnt transcriptional program [9,10]. 

**Figure 1:**
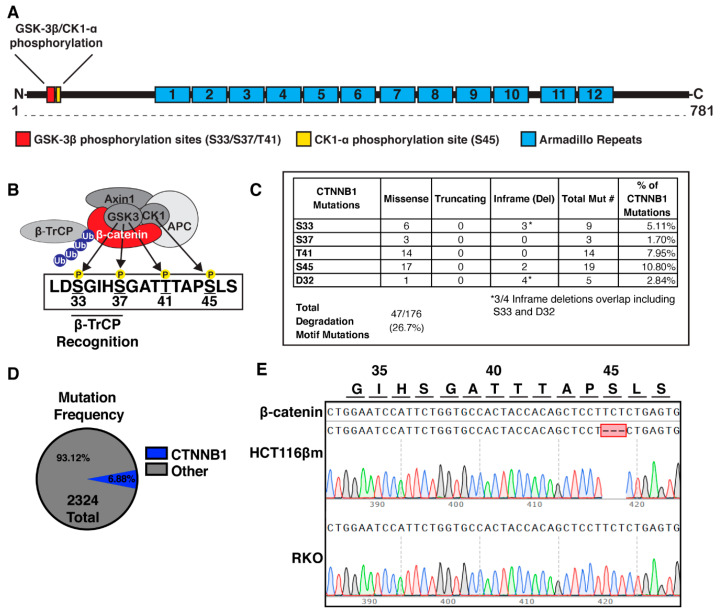
β-catenin phospho-regulation by the β-catenin destruction complex. (**A**) Diagram of β-catenin structure, indicating the N-terminal GSK-3β phosphorylation sites (Ser33/Ser37/T41), the CK1-α phosphorylation site (Ser45), and the 12 armadillo repeats. (**B**) Schematic of the β-catenin destruction complex composed of Axin, Adenomatous Polyposis Coli (APC), GSK-3β, and CK1-α, which bind and phosphorylate β-catenin at Ser45, Thr41, Ser37, and Ser33. Phosphorylation at Ser33/Ser37 creates a β-TrCP recognition site. (**C**) Mutation frequency of destruction complex phosphorylation sites among *CTNNB1* mutations. (**D**) Mutation frequency of *CTNNB1* among 2324 colorectal cancer patients using cBioPortal. (**E**) Sanger sequencing and alignment of *CTNNB1* around the destruction complex phosphorylation sites in HCT116βm and RKO colon cancer cell lines.

In canonical Wnt signaling, β-catenin destruction is initiated through a dual-kinase mechanism. First, CK1-α phosphorylates Ser45 of β-catenin (Figure 1A,B) [8,11]. Next, phospho-Ser45 primes GSK-3β activity, which typically requires a phospho-Ser or threonine and the S/T-X-X-X-pS/pT motif [12,13,14]. Three sites on β-catenin are then primed and phosphorylated hierarchically, beginning with Thr41, then Ser37, and finally Ser33, as each phosphorylating event primes the next (see Figure 1). Phosphorylation of Ser33 and Ser37 generates a WD40-like binding site for β-TrCP, the substrate recognition subunit of the E3 ubiquitin ligase SCF^β-TrCP^ which ubiquitinates β-catenin and marks it for degradation by the proteasome [7,15,16,17,18]. A mutation that eliminates any one of the phosphorylation sites is thought to stabilize β-catenin, making it resistant to destruction and able to activate downstream Wnt signaling.

Mutations in the tumor suppressor *Adenomatous Polyposis Coli* (*APC*) occur early in the development of over 80% of CRCs. The vast majority of *APC* mutations lead to the expression of truncated APC protein that retains some ability to interact with and regulate β-catenin. The “just right” model rationalizes the limited range of APC truncations observed in CRCs as facilitating a precise level of β-catenin for optimal cellular proliferation—not too much or too little [19]. In addition to a scaffolding function, other APC activities have been suggested to contribute to Wnt signaling. For example, APC can interact with nuclear β-catenin, leading to repression of Wnt target genes through several proposed mechanisms: providing access to the transcriptional corepressor CtBP or E3 ligase β-TrCP, sequestration of β-catenin from the transcriptional coactivator LEF-1/TCF, or facilitating β-catenin’s nuclear export [20,21,22,23,24]. APC can maintain interaction with β-catenin following Wnt stimulation and appears critical for trafficking the destruction complex to the Wnt receptors [25,26]. Additionally, APC has been postulated to promote β-catenin ubiquitination. APC truncation commonly found in human CRCs renders cells unable to appropriately ubiquitinate β-catenin and target it for proteasomal degradation [25,27,28]. A region of APC just C-terminal to sites of common truncations appears to be sufficient for the rescue of β-catenin ubiquitination [25,28].

CRCs without *APC* mutations commonly have mutations in genes encoding other components of the Wnt pathway. The key downstream effector molecule in Wnt signaling, the β-catenin gene, *CTNNB1,* is mutated in ~12% of CRCs lacking an *APC* mutation (Table S1). It is curious why mutations that protect β-catenin from degradation are not more prevalent in CRC, as this would be a direct path to β-catenin mediated transcription. It is possible that mutations that completely stabilize β-catenin do not support cell viability. In this event, β-catenin mutations may also act in a “just-right” manner to precisely tune the amount of β-catenin for optimal levels of Wnt activation. Another, not mutually exclusive, possibility is that destroying β-catenin is not the only critical tumor-suppressive role for APC, and that additional APC-mediated processes such as cytoskeletal arrangement, β-catenin localization, and cellular orientation during cell division must be affected to initiate adenoma formation. 

Mutations that eliminate the GSK-3β or CK1-α phosphorylation sites are reported to “stabilize” β-catenin, making it resistant to regulation by the destruction complex and thus resistant to regulation by Wnt signaling [5]. Cells expressing β-catenin containing a Ser45 deletion or S33Y substitution are reported to display constitutively active Wnt signaling [29]. HCT116 cells, which express two versions of β-catenin (a wild-type and a Ser45-deleted) show elevated Wnt reporter activity when treated with Wnt, however, this was explained by the retained ability to regulate the wild-type β-catenin [25]. While the prevailing notion has maintained that oncogenic *CTNNB1* mutations result in an abolished response to Wnt, new evidence suggests otherwise. In hepatocellular carcinoma (HCC) cells, Rebouissou and colleagues reported that S45 mutations are only weakly activating and they concluded that S45 mutation alone is not sufficient to drive liver tumorigenesis [30]. Our own analysis of 351 liver cancers using cBioPortal confirmed that patients with S45 mutations showed elevated expression of Wnt target genes *Axin2*, *GLUL*, and *LGR5*, but not to the levels seen in patients with mutations in D32-S37 (Table S2). We recently demonstrated that colon cancer cells expressing only mutant β-catenin (Ser45del, termed β-catΔS45) still show the redistribution of both the destruction complex and β-catΔS45 toward a localized Wnt ligand [26]. Phosphorylation at the GSK-3β sites (Ser33/Ser37/Thr41) was previously reported to occur in the absence of Ser45 [31]. These results indicate that the presence of β-catΔS45 does not render cells completely unresponsive to Wnt and raises additional questions about the mechanism underlying β-catenin regulation. 

Here, we sought to further test the “just-right” model of β-catenin mutation proposed for hepatocellular carcinoma by Rebouissou et al. by examining the effect of a β-catΔS45 mutation on the Wnt response in HCT116βm cells which harbor a single *CTNNB1* allele encoding a Ser45 deletion [32]. Ser45 modifications are seen in 10.8% of CRCs with β-catenin alterations and are considered to be stabilizing (Figure 1C). We demonstrate for the first time that HCT116βm cells accumulate β-catΔS45 when treated with Wnt3a or when depleted for APC and also display increased downstream Wnt transcriptional activation. However, β-catΔS45 nuclear translocation is only elevated in cells depleted for APC and not by Wnt3a treatment alone. We also find that β-catΔS45 is phosphorylated on the S33/S37/T41 residues, albeit somewhat less than wild-type β-catenin. It has been proposed that “just-right” signaling could result from *CTNNB1* mutation as well as *APC* mutation [33]. Our work demonstrates that β-catΔS45 is regulated by the destruction complex and responds to Wnt by accumulating and becoming more active in promoting Wnt target gene transcription. Further, these results implicate additional roles for APC in β-catenin regulation beyond those as a destruction complex scaffold. 

## 2. Results

### 2.1. Phosphorylation Sites Important For Β-Catenin Destruction Are Mutated in a Subset of Colorectal Cancers 

Over-active Wnt signaling has been linked to many malignancies. In liver and endometrial cancer, *CTNNB1* mutations are common while *APC* mutations are rare [34]. In the majority of CRCs, mutations in *APC*, a key member of the β-catenin destruction complex, predominate. In CRCs without *APC* mutation, the Wnt pathway is often activated by other means, such as mutation of another pathway component. To assess the frequency *of CTNNB1* mutation in CRCs, we utilized four datasets from cBioPortal (DFCI, Genentech, MSKCC, and TCGA) and found that β-catenin mutations occurred in 160/2324 of patients (6.88%; Figure 1D) [4,35,36,37,38,39]. Of these β-catenin mutations, 47/176 were in the degradation motif sites (Figure 1C). Mutations within exon 3 of *CTNNB1* are considered drivers of tumorigenesis and account for 57/176 of the analyzed mutations [40]. Additionally, 91/176 mutations (51.7%) occurred within the armadillo repeat regions, which are thought to interact with APC, Axin, and LEF-1 [41,42,43]. We note that mutations outside of exon 3 may be passenger mutations and also find that truncating events that are unlikely to activate Wnt/β-catenin signaling account for 32/176 mutations. The “just-right” signaling hypothesis has been proposed as a means for the cell to regulate levels of Wnt activity through mutation of the *APC* gene. We wondered if mutations of the effector protein, β-catenin, also invoke a similar “just-right” response, allowing a specific level of Wnt regulation. 

### 2.2. Generation of a Novel Anti-APC Ιgy Antibody

Current commercially available antibodies for the analysis of APC have limited applications and specificity [44,45]. To expand the repertoire of antibody species, we generated a chicken polyclonal antibody using the same central region of APC (amino acids 1001–1326) which we had successfully used to generate rabbit antisera [46]. The new IgY antibody was purified from yolk extracts and tested by Western immunoblot (Figure S1). The major band detected by the purified antibody migrated with an apparent molecular weight of 310 kDa with only faint signals for the smaller sized bands. When compared against a commercially available APC antibody, our chicken antibody shows a robust signal. Using siRNA to efficiently knockdown APC, we confirm that the 310 kDa band is reduced upon APC-depletion. This new tool will allow specific detection of APC, simultaneously with other proteins detected using mouse or rabbit antibodies. 

### 2.3. Β-Catδs45 Associates With a Locally-Applied Wnt-3a Ligand

**Figure 2:**
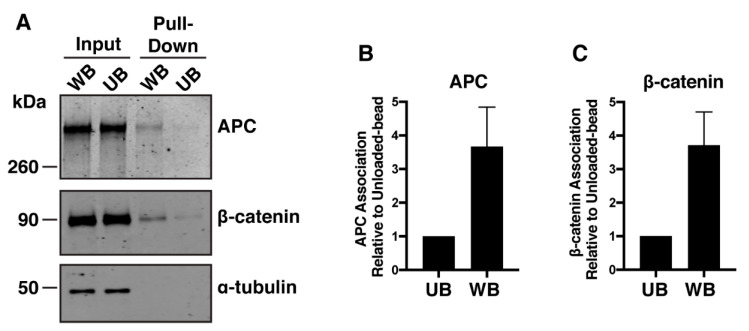
Wnt-beads pull-down APC and β-catΔS45. (**A**) HCT116βm cells treated with Wnt-beads or Unloaded-beads were lysed and proteins were pulled-down with the beads. Both APC and β-catΔS45 were detected in the Wnt-bead pull-down but not the Unloaded-bead pull-down. (**B**) Quantification of APC pulled-down by Wnt-beads compared to Unloaded-beads from three independent experiments. (**C**) Quantification of β-catΔS45 pulled-down by Wnt-beads compared to Unloaded-beads from three independent experiments. Protein levels that were pulled-down by beads were divided by the respective input protein levels and normalized to the Unloaded-bead to demonstrate fold change. Error bars, standard deviation (SD)Full blots, Figure S2).

Using immunofluorescence microscopy, we previously established that β-catΔS45 localizes toward a Wnt cue in HCT116βm cells [26]. We initially verified that a single allele encoding β-catenin is present in HCT116βm cells by performing Sanger sequencing, using RKO cells, another CRC cell line, which express only wild-type β-catenin as a control (Figure 1E). To test for a physical interaction between Wnt and β-catΔS45, Wnt3a-conjugated beads were applied to HCT116βm cells and then used to “pull down” associated proteins from cell lysates (Figure 2A). APC associated with Wnt-beads more than with the Unloaded-beads (Figure 2B). β-catΔS45 also associated more with the Wnt-beads than with the Unloaded-beads (Figure 2C). These data demonstrate that a core component of the destruction complex and a “stabilized” β-catenin both respond to a Wnt3a cue by localizing to the membrane, presumably through interactions with Frizzled and LRP5/6 coreceptors. 

### 2.4. Wnt3a Exposure or APC-Depletion Increases Level of Β-Catδs45 Protein

**Figure 3:**
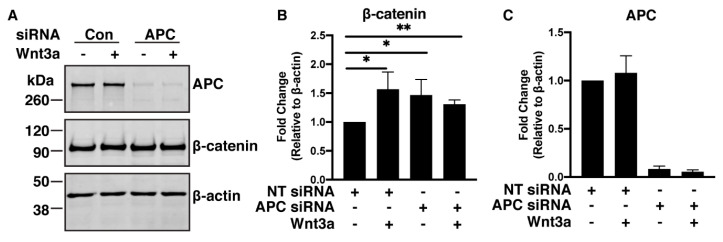
β-catΔS45 accumulates upon Wnt-3a treatment or APC-depletion. (**A**) Western blot of HCT116βm cells transfected with control-siRNA or APC-siRNA and treated in the presence or absence of 125 ng Wnt3a. (**B**) Relative fold-change of β-catenin relative to β-actin and normalized to control-siRNA without Wnt. Data averaged from four independent experiments. Error bars, SD; Statistical analysis by *t*-test: * *p* < 0.05; ** *p* < 0.01. (**C**) Quantification of APC fold-change relative to β-actin and normalized to control-siRNA without Wnt.

We previously reported that β-catenin destruction complex localization toward a Wnt cue was APC-dependent and appeared to correlate with an increased level of β-catenin in cells harboring fully intact Wnt signaling pathways [26]. Whether Wnt influences β-catΔS45 protein levels has yet to be determined. The β-catΔS45 protein expressed in HCT116βm cells lacks the site of CK1-α phosphorylation and is therefore generally assumed to be compromised for phosphorylation by GSK-3β and subsequent proteasome-mediated destruction. Notably, treatment with Wnt3a resulted in a 1.57-fold increase in total β-catΔS45 compared to untreated cells (Figure 3A–C). This unexpected result indicates that HCT116βm cells at least partially respond to Wnt by further stabilizing β-catΔS45 and therefore, β-catenin degradation can occur independently of Ser45. We efficiently depleted 90–95% of APC in HCT116βm cells with small interfering RNA (siAPC; Figure 3A,C). This APC-depletion led to a 1.47-fold increase in total β-catΔS45 protein level, while APC-depletion combined with Wnt3a treatment led to a 1.31-fold increase (Figure 3B). All of these increases were significant when compared to control-siRNA-treated cells (NT siRNA). These data indicate that cells exposed to Wnt ligand are able to further increase β-catΔS45 levels and that APC participates in β-catΔS45 destruction. Because APC-depletion together with Wnt treatment did not result in β-catΔS45 protein levels greater than either condition alone, it seems likely that these two components function in the same pathway to control β-catΔS45 protein levels. 

### 2.5. Wnt Signaling Is Activated in HCT116βm Cells Following APC-Depletion or Wnt3a Exposure

**Figure 4:**
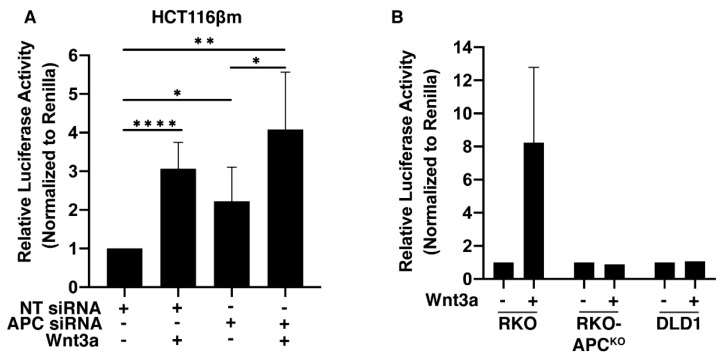
The Wnt transcriptional program is induced in HCT116βm upon Wnt-3a treatment or APC-depletion. (**A**) HCT116βm cells were cotransfected with pTOPflash and pRenilla luciferase reporter plasmids as well as control-siRNA or APC-siRNA, and subsequently treated with 125 ng Wnt3a for 24 h. Data averaged from seven individual experiments. Relative luciferase activity determined by the TOPflash/Renilla ratio of each group, followed by normalization to the control. Error bars, SD; Statistical analysis by *t*-test: * *p* < 0.05; ** *p* < 0.01; **** *p* < 0.0001. (**B**) TOPflash reporter assay in RKO, RKO-APC^KO^, and DLD-1 colorectal cancer cell lines. Data for RKO averaged from four individual experiments, and RKO-APC^KO^ and DLD1 are from one experiment. Error bars, SD.

In cells with an intact Wnt signaling pathway, cellular β-catenin accumulation is followed by β-catenin’s nuclear translocation and interaction with the transcription cofactor TCF-4 to activate Wnt target genes. HCT116βm cells harbor an activated Wnt pathway, demonstrated through increased TOPflash compared to isogenic cells only expressing wild-type β-catenin [32]. We wondered if the increased level of cellular β-catenin following Wnt exposure or APC-depletion would result in further increases in nuclear β-catenin activity. To test this, we cotransfected HCT116βm cells with TOPflash Wnt luciferase reporter plasmid and then depleted APC with siRNA, in the presence or absence of Wnt3a. Wnt3a treatment resulted in a three-fold increase and APC-depletion led to a 2.2-fold increase in Wnt reporter activity (Figure 4A). Unexpectedly, cells both depleted for APC and exposed to Wnt3a displayed a four-fold increase in Wnt reporter activity (Figure 4A). In RKO cells, which possess an intact Wnt signaling pathway, Wnt treatment resulted in an 8.2-fold increase in Wnt reporter activity (Figure 4B). However, in RKO cells with CRISPR/Cas9-deleted APC or in DLD1 cells that express endogenous truncated APC, Wnt3a presentation had no effect on Wnt reporter activity (Figure 4B). 

Combined, these data indicate that β-catΔS45 is still regulated by Wnt signaling, despite being able to evade Ser45 phosphorylation by CK1-α. Interestingly, cells with APC knock-out or mutant APC are resistant to further Wnt reporter activation, presumably due to maximal levels of pathway activation (Figure 4B) [25]. Yet, HCT116βm cells containing a stabilized β-catΔS45 are responsive to APC-depletion, even more responsive to Wnt addition, and show the most Wnt reporter activation when APC-depletion is combined with Wnt stimulation. This finding suggests that Wnt3a presentation and APC loss may stimulate Wnt reporter activity in an additive manner. This additive response may indicate the involvement of multiple pathways.

### 2.6. β-catΔS45 Increases Nuclear Localization Upon APC Loss, But Not Upon Wnt Exposure 

**Figure 5:**
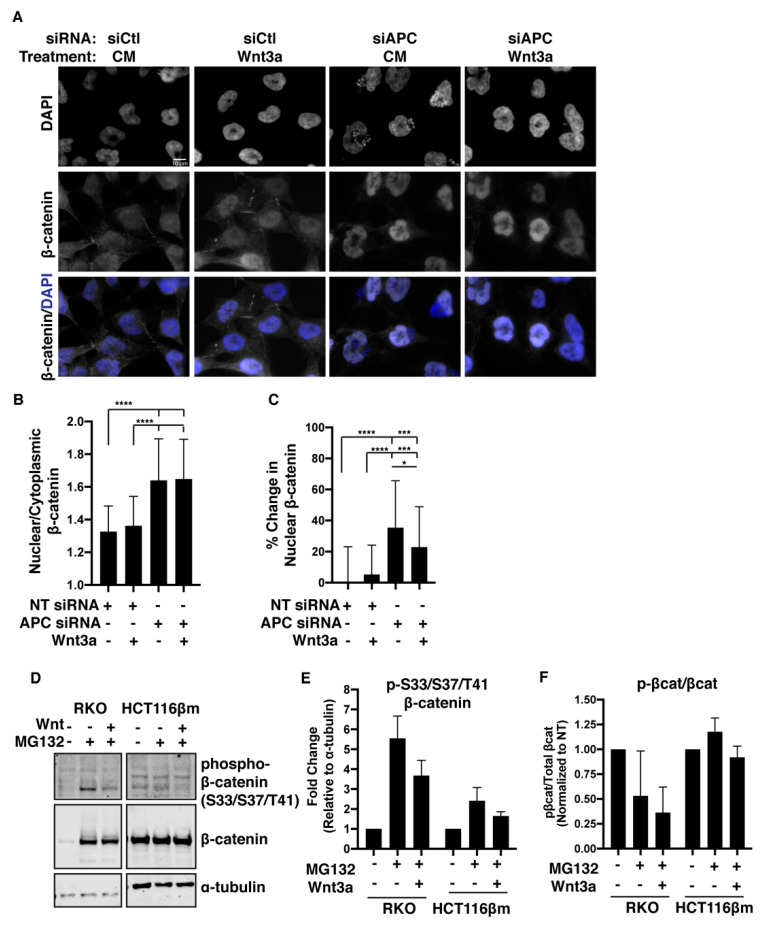
β-catΔS45 nuclear localization is controlled by APC and continues to be susceptible to GSK-3β phosphorylation and proteasomal degradation. (**A**) HCT116βm cells transfected with control-siRNA or APC-siRNA were treated with 125 ng Wnt3a and β-catenin localization was visualized by immunofluorescent detection. (**B**) Ratio of nuclear to cytoplasmic β-catenin quantified from immunofluorescent staining. Data representative of at least 36 individual cells per group (from two independent experiments). Error bars, SD; Statistical analysis by *t*-test: * *p* < 0.05, *** *p* < 0.001, **** *p* < 0.0001. (**C**) Percent change in nuclear β-catenin. Percent of nuclear β-catenin was calculated by dividing nuclear β-catenin by total β-catenin. Using nuclear β-catenin percentage, the percent change was calculated by comparing it to control. (**D**) RKO and HCT116βm cells were treated with 10 mM MG132 in the presence or absence of 25 ng/mL Wnt3a for 4 h prior to lysis. Western blot utilizing anti-phospho-Ser33/Ser37/Thr41-β-catenin or anti-β-catenin antibodies. (**E**) Quantification of the fold change of Ser33/Ser37/Thr41-phosphorylated β-catenin. Data averaged from two independent experiments. Error bars, SD. (**F**) Quantification of the ratio of phosphorylated-β-catenin to total β-catenin in RKO and HCT116βm cells following treatment with MG132 +/− Wnt3a. Data averaged from two independent experiments. Error bars, SD.

APC-depletion has revealed a mechanism to regulate β-catΔS45 activity that appears distinct from that of Wnt stimulation. To explain this, we turned to other potential APC functions. Notably, APC is reported to be involved in sequestration, trafficking, and nuclear-cytoplasmic shuttling of proteins as well as Wnt-induced membrane localization of the destruction complex. We therefore considered the possibility that APC aids in the sequestration or trafficking of β-catΔS45, despite the ability of β-catΔS45 to evade destruction-complex-mediated Ser45 phosphorylation. We found that cells treated with Wnt displayed no changes in the ratio of nuclear to cytoplasmic β-catΔS45 compared to control cells (Figure 5A,B). However, upon APC-depletion, β-catΔS45 shifted into the nucleus, displaying an increased nuclear/cytoplasmic ratio and increased level of nuclear β-catenin compared to control (Figure 5A–C). Altered nuclear morphology observed in APC-depleted cells is consistent with previous reports that APC loss can lead to apoptosis [47,48]. The ratio of nuclear to cytoplasmic β-catΔS45 seen with APC-depletion did not further increase with added Wnt treatment (Figure 5B). Although still more nuclear than in control cells, the percent of change in nuclear β-catΔS45 in APC-depleted cells that were also treated with Wnt was significantly less than in cells only depleted of APC (Figure 5C). These data indicate that regulation of a “stabilized” β-catΔS45 is not merely through a degradation mechanism, but also may involve cytoplasmic sequestration or nuclear export, facilitated by APC. 

Assessment of β-catΔS45 nuclear and cytoplasmic localization showed that Wnt treatment alone does not affect nuclear translocation. However, APC-knockdown or Wnt treatment plus APC-knockdown caused a dramatic increase in nuclear β-catΔS45 compared to control. Of note, through assessment of β-catenin level (Figure 3), β-catΔS45 activity (Figure 4), and β-catΔS45 nuclear translocation (Figure 5), we found that increases in the level and nuclear localization of β-catΔS45 did not always translate into increased activity. Using these different data sets, we normalized the TOPflash values to the level of nuclear β-catΔS45 to estimate activity per unit of nuclear β-catΔS45. This analysis revealed that, though there was little change in nuclear β-catΔS45 levels, Wnt treatment alone resulted in a near doubling of the β-catΔS45 activity (1.92-fold) compared to untreated cells. In contrast, APC-depletion resulted in more β-catenin protein and nuclear localization but did not alter the activity per β-catΔS45 unit (1.05-fold) compared to untreated cells. The combination of APC-depletion and Wnt treatment resulted in elevated β-catenin protein and nuclear localization, similar to that observed with only APC-depletion. Finally, the activity per β-catΔS45 unit was much higher (2.5-fold) in combination-treated cells than that of untreated cells or only APC-depleted cells. 

### 2.7. β-catΔS45 Is Phosphorylated at the GSK-3β Sites and Is Susceptible to Proteasomal Degradation

Solely considering the destruction complex, it is perplexing that APC-depletion or Wnt exposure would impact the protein level of a “stabilized” β-catenin. Previously, it was reported that β-catΔS45 is phosphorylated at the GSK-3β sites Ser33/Ser37/Thr41 [31]. However, it was unknown whether β-catΔS45 is also degraded by the proteasome. We detected more total β-catenin and phospho-Ser33/Ser37/Thr41-β-catenin when RKO cells were treated with an MG132 proteasome inhibitor (Figure 5D,E). This was expected, since RKO cells have an intact β-catenin destruction complex. The ratio of p-β-catenin to total β-catenin decreased in RKO cells treated with MG132 (Figure 5F). This decrease is potentially due to the large increase in total β-catenin level (~14-fold), and may reflect saturated destruction complexes unable to phosphorylate all of the accumulated β-catenin, as previously proposed [25]. We also detected phospho-β-catΔS45 in HCT116βm cells, thus confirming previous reports (Figure 5D) [31]. MG132 treatment resulted in increased total β-catΔS45 and phospho-β-catΔS45 levels, indicative of proteasomal degradation (Figure 5E). However, we did not observe a decreased ratio of p-β-catΔS45/β-catΔS45 in HCT116βm cells treated with both MG132 and Wnt (Figure 5F). Together, these data support a proposed mechanism in which cells containing a deletion of the CK1-α phosphorylation site are able to maintain some β-catenin regulation through the potential alternative priming of GSK-3β-mediated phosphorylation and through proteasomal degradation. Of note, this ability to respond to Wnt ligand distinguishes β-catΔS45-mutant colorectal cancer cells from the APC-mutant colorectal cancer cells (Figure 4B). 

### 2.8. APC Truncation But Not β-Catenin Mutation Results in Elevated Wnt Target Gene Expression in Human Colorectal Cancers

**Figure 6:**
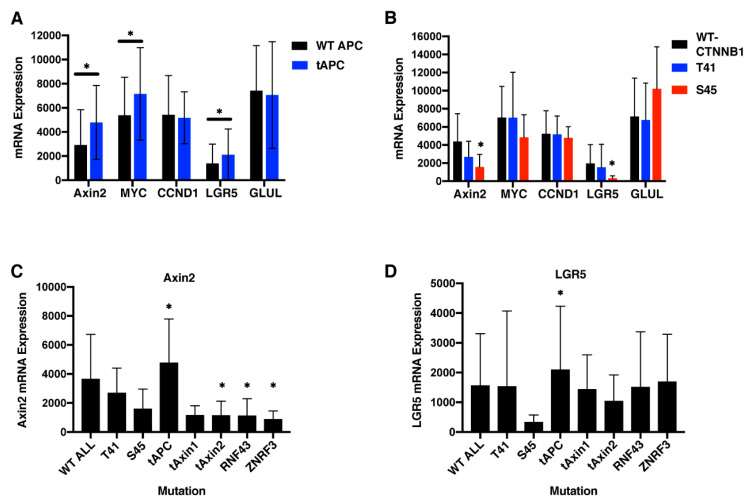
Wnt target gene activation in colorectal cancers with truncating APC mutations. Wnt target gene mRNA expression was determined from 524 colorectal cancer patients using cBioPortal and is displayed as RSEM. *CCND1* encodes CyclinD1 and *GLUL* encodes Glutamate–Ammonia Ligase. (**A**) mRNA expression in 374 patients with truncating “driver” *APC* mutations and 150 with wild-type *APC* or missense mutations (all included in the wild-type category). (**B**) mRNA expression for 491 patients with wild-type *CTNNB1*, 7 with Ser45 mutations, and 6 with Thr41 mutations. For (**C**) and (**D**) 426 colorectal cancer patient samples were queried for levels of Wnt target AXIN2 or *LGR5* RNA, respectively. Samples were categorized by mutation status of *CTNNB1* or truncating driver mutations in β-catenin regulatory genes. WT ALL samples had no driver mutations in any Wnt-related category queried. Error is presented as SD. * indicates *p*-value calculated with paired student *T*-test < 0.0005 in (**A**) and < 0.05 in (**B**–**D**) for each pair combination. For (**C**) and (**D**), n values of each group were as follows: WT All (101), T41 (6), S45 (7), t*APC* (374), t*Axin1* (5), t*Axin2* (19), *RNF43* (36), and *ZNRF3* (8).

Having demonstrated that β-catΔS45 still shows evidence of regulation by Wnt and APC, we turned our attention back to human colorectal cancer patient samples. Using cBioPortal, we queried the TCGA (PanCancer Atlas) dataset for expression of Wnt target genes in colon cancers with various categories of *APC* or *CTNNB1* mutations. mRNA levels for demonstrated Wnt targets *Axin2*, *Myc*, and *Lgr5* were significantly elevated in CRC tumors with *APC* mutations that result in protein truncation vs. samples lacking these truncating *APC* mutations, designated as wild-type (Figure 6A). Therefore, expression patterns for these three genes can provide a surrogate marker for β-catenin signaling in the patient tissue samples. In contrast, *CCND1* and *GLUL*, which have also been described as Wnt targets, showed no expression changes that correlated with *APC* status.

Using a similar approach, sample groups were established based on *CTNNB1* status (Figure 6B). There were seven samples with Ser45 mutations (deletions or substitution of Phe or Pro) and six with T41 mutations (Ala or Ile substitution). In liver cancers, these mutations were categorized as having “weak” and “moderate” β-catenin activity, respectively [30]. Other *CTNNB1* mutations were not present in enough patient samples to analyze. Relative to samples with wild-type *CTNNB1*, none of the Wnt target gene markers showed significant upregulation in either the Ser45- or Thr41-mutant group. Rather, the mRNA level for the β-catenin activity reporters that were elevated with *APC* truncating mutation was either unchanged or decreased in patients with *CTNNB1* mutations when compared to patients with wild-type *CTNNB1*. Over 70% of the colorectal cancer patient samples with wild-type *CTNNB1* displayed truncating *APC* mutations and another 9% displayed mutations in one or more other genes involved in β-catenin destruction (*Axin1*, *Axin2*, *RNF43*, and *ZNRF3*). To ensure that mutations in these other β-catenin regulators were not influencing Wnt target gene expression in our “wild-type” samples presented in Figure 6A,B, the RNA levels were reanalyzed taking into account the status of these various Wnt regulatory genes (Figure 6C,D). Once again, the mRNA levels for β-catenin activity reporters *AXIN2* and *LGR5* were either unchanged or decreased in patients with *CTNNB1* mutations when compared to patients that were wild-type for all of these β-catenin regulator genes. Though these results were consistent with our in vitro data, they contradict results from a similar analysis of liver cancers [30] and (Table S2). We conclude that in colon cancers, the Ser45 mutation does not appear to increase Wnt signaling and that the effects of *CTNNB1* mutations are not the same in the liver and colon.

## 3. Discussion

The Wnt/β-catenin pathway is typically described as signaling in a linear manner; Wnt ligand binds the Frizzled and LRP5/6 coreceptors, leading to inhibition of the β-catenin destruction complex and subsequent β-catenin stabilization, accumulation, and nuclear translocation. Mutation to a downstream component such as APC or β-catenin is thought to activate the pathway and render it unresponsive to regulation by upstream pathway components. The results presented here demonstrate that “downstream” components of the Wnt pathway can continue to be regulated by “upstream” components despite the presence of a stabilizing mutation. In the context of APC mutations, a “just-right” signaling hypothesis has been proposed in which APC mutations occur to allow a specific level of β-catenin that supports tumorigenesis. Our results support the notion that β-catenin itself may also be prone to just-right mutations that maintain some regulation of the β-catenin protein, limiting its accumulation and activity. As an initial proof of concept, we used the HCT116βm cell line which harbors a deletion of β-catenin Ser45, to assess the Wnt/Receptor/β-catΔS45 interaction and the changes in β-catΔS45 protein levels, activity, and localization upon Wnt3a stimulation or APC loss.

In agreement with our previous immunofluorescence data [26], we demonstrate that both β-catΔS45 and APC pull-down with a Wnt-bead, indicative of the interaction between Wnt ligand, receptor, β-catΔS45, and APC. Surprisingly, we find that Wnt3a treatment, APC knock-down, or treatment plus knockdown, results in elevated β-catΔS45 protein levels as well as increased TOPflash Wnt reporter activity. APC-depletion results in predominantly nuclear β-catΔS45, whereas Wnt treatment alone does not change the nuclear localization of β-catΔS45. Moreover, we confirm that β-catΔS45 can be phosphorylated at Ser33/Ser37/Thr41 and find that β-catΔS45 is regulated by the proteasome. Finally, we provide evidence that in colorectal cancer patient tissue, *CTNNB1* mutations that eliminate Ser45 or Thr41 do not result in elevated β-catenin activity as assessed by Wnt target gene expression.

We propose the following expansion of the current Wnt signaling mechanism to explain our findings: (1) Wnt signaling leads to a secondary effect that further activates β-catenin in the nucleus, essentially amplifying the signal. An example of this would be that *LEF*/*TCF* is a Wnt target gene [49,50]. (2) APC inhibits the activity of this Wnt-induced β-catenin inducer. (3) APC promotes cytoplasmic localization of β-catenin, perhaps through cytoplasmic sequestration or nuclear to cytoplasmic shuttling. Our results are inconsistent with the role of APC in sequestering nuclear β-catenin or facilitating the nuclear import of β-catenin. (4) Wnt can also inhibit nuclear localization of β-catenin, independent of APC. (5) *CTNNB1* mutations to Thr41 or Ser45 in human CRC do not confer elevated Wnt signaling. 

Our work is in agreement with previously published results by our lab and others, demonstrating that the interaction of APC with nuclear β-catenin leads to repression of Wnt target genes [20,21,23,24]. Further, it is intriguing that β-catΔS45 continues to be phosphorylated at GSK-3β sites and degraded by the proteasome, albeit less efficiently than wild-type β-catenin. The deletion of Ser45 may place Ser47 in close enough proximity (within five residues upon S45del) to T41 to act as a priming site for β-catenin phosphorylation [51]. Suggestive of alternative mechanisms for GSK-3β phosphorylation, S33/S37/T41 phosphorylation is also observed in LS174T cells containing a S45F substitution [25,31]. Finally, comparing our Wnt target gene mRNA expression analysis in human colon cancer tissue to similar analyses in liver cancer [30] and Table S2 reveals that Wnt signaling and the consequences of *CTNNB1* mutation are not the same in cancers of different tissues. 

Colorectal cancer is generally assumed to be promoted by Wnt signal activation. Here, we provide insight into the mechanism by which β-catenin is regulated in the Wnt-on or Wnt-off states and also demonstrate that mutant β-catenin can be regulated by the Wnt pathway components. We find that *APC*-mutant cells are unresponsive to additional Wnt signaling, whereas cells expressing mutant β-catenin are responsive to extracellular Wnt ligand. It is likely that complete stabilization of β-catenin in colon epithelia would result in too much β-catenin protein and activity, thus impairing cell viability and potentially inducing apoptosis as previously demonstrated in mouse gut [48] and human epidermis [52]. Our finding that the Wnt/β-catenin signaling pathway does not act in a strictly linear fashion emphasizes the importance of a therapeutic strategy that targets multiple aspects of the pathway. Finally, we provide evidence that β-catΔS45 is still susceptible to regulation and that this mutation may act to provide “just-right” signaling for optimal tumorigenic capability in the colon.

## 4. Materials and Methods 

### 4.1. Cell Culture and Treatments

HCT116βm, RKO, DLD1, and RKO-APC^KO^ cells were cultured in DMEM (with L-Glutamine and 4.5 g/L Glucose; without Sodium Pyruvate) supplemented with 10% fetal bovine serum (FBS) and were maintained at 37 °C and 5% CO_2_. Wnt treatment was performed by adding recombinant Wnt-3a (Peprotech, Rocky Hill, NJ, USA; #315-20) at the indicated concentration/time prior to cell lysis or immunofluorescence analysis. For siRNA-mediated inhibition, HCT116βm cells were transfected using Lipofectamine 3000 (Invitrogen, Carlsbad, CA, USA) according to the manufacturer’s instructions with 37.5 nM of each siRNA targeting human APC (Smartpool siRNAs 1-3: Dharmacon, Lafayette, CO, USA) or nontargeting siControl siRNA (Dharmacon). Cell media was changed one day following siRNA transfection, and cells were grown 48 h prior to Wnt treatment. MG-132 treatment was performed by the addition of 10 mM MG-132 to a final concentration of 10 μM in cell media for 4 h.

HCT116βm were derived from the parental HCT116 cell line (ATCC, Manassas, VA, USA) and kindly provided by Bert Vogelstein [32]. We received HCT116βm from Bert Vogelstein (who originally received HCT116 cells from ATCC) and passaged two times into Dulbecco’s Modified Eagle Medium (DMEM)+10%FBS prior to preparing cell stocks. RKO and DLD1 cells were received from ATCC and passaged two times into DMEM + 10% FBS prior to preparing cell stocks. RKO-APC^KO^ cells were derived from the parental RKO cell line (ATCC) and kindly provided by Ethan Lee [53]. We passaged them two times into DMEM+ 10% FBS prior to preparing cell stocks.

### 4.2. Analysis of CTNNB1 Mutation Frequency and mRNA Expression of Wnt Target Genes

Mutation frequency of the *CTNNB1* gene was assessed using the cBioPortal [35,36]. Colorectal adenocarcinoma datasets from DFCI (Cell Reports 2016), Genentech (Nature 2012), MSKCC (Cancer Cell 2018), and TCGA (PanCancer Atlas) were queried for the *CTNNB1* and *APC* genes [37,38,39,54]. Of the 1287 total cases, 1225 had mutations data and were utilized for mutation frequency and mutual exclusivity determinations. The TCGA (PanCancer Atlas) dataset also contained RNAseq data which were queried for expression of Wnt target genes in colon cancers with various categories of *CTNNB1* mutations or “driver” mutations in *APC* or other β-catenin regulatory factors. Values were provided as RSEM (RNA-Seq by Expectation Maximization).

### 4.3. CTNNB1 Sanger Sequencing and Alignment

HCT116βm and RKO cells were harvested and DNA extraction was performed using the Qiagen (Hilden, Germany) DNeasy Blood and Tissue Kit according to the manufacturer’s instructions. PCR amplification was performed using the following primers: 5′-cctcctaatggcttggtgaa-3′; 5′-caggacttgggaggtatcca-3′. Following amplification, PCR products were gel-purified and sequenced by Genewiz (South Plainfield, NJ, USA). Trace files and sequence alignment were analyzed using SnapGene, version 4 (Insightful Science). 

### 4.4. Immobilization of Wnt Protein

Wnt3a was immobilized onto Dynabeads as described previously [55]. Briefly, 2.8 μm Dynabeads M-270 Carboxylic Acid (Invitrogen) were activated by NHS/EDC (Sigma, St. Louis, MO, USA, 50 mg/mL each in cold 25 mM MES pH 5) then washed three times with cold 25 mM, pH5 5 2-(*N*-morpholino)ethanesulfonic acid (MES) buffer. Wnt immobilization was performed by diluting 0.5 μg of purified Wnt3a protein in cold MES buffer and incubated at room temperature (RT) for 1 h. To quench nonreactive carboxylic acid groups, beads were incubated with 50 mM Tris pH 7.4 at RT for 15 min. Beads were washed twice in phosphate-buffered saline (PBS) pH 7.4 before final resuspension in 400 μL PBS/0.5% BSA and stored at 4 °C. Unloaded-beads were prepared in parallel by incubating 1 h in MES without Wnt. Wnt3a activity following bead immobilization was verified using a TOPflash luciferase reporter assay [56]. 

### 4.5. Immunoblotting

Cells were washed 1x in PBS prior to harvesting in preheated, high-salt sample lysis buffer (20% glycerol, 2% sodium dodecyl sulfate (SDS), 30% 10X PBS, 2.5% β-mercaptoethanol). Scraped cells were transferred to Eppendorf tubes, heated at 95 °C for 1 min, pulled through an insulin syringe three times, and heated again. Samples were separated on 7.5% SDS-PAGE (Bio-Rad, Hercules, CA, USA; TGX FastCast Acrylamide Kit) using Tris-Glycine running buffer and transferred to a nitrocellulose membrane (GE) with a 0.45 μm pore size. Antibodies were diluted in Odyssey Blocking Buffer TBS (LI-COR) as follows: anti-APC-M2 Chicken pAb (1:2000), anti-β-catenin mouse mAb (1:1000), anti-phospho-Ser33/Ser37/Thr41-β-catenin rabbit pAb (Cell Signaling Technology, Danvers, MA, USA; 1:500), anti-α-tubulin DM1A mouse mAb (Santa Cruz Biotechnology, Dallas, TX, USA; 1:1000), anti-β-actin mouse mAb (Sigma, 1:1000), and IRDye 680LT and 800CW anti-rabbit, anti-mouse, or anti-chicken secondary antibodies (1:150,000). Immunoblots were imaged on an LI-COR Odyssey CLx imaging system. 

### 4.6. Wnt-Bead Pull-Down

Cells were grown in 6-well tissue culture plates and treated with 40 μL Unloaded-beads or Wnt-beads for 4 h. Following bead treatment, cells were briefly washed in 1x PBS prior to lysis in 200 μL lysis buffer (150 mM NaCl, 30 mM Tris pH 7.5, 1 mM EDTA, 1% Triton X-100, 10% glycerol, 0.1 mM PMSF, 0.5 mM DTT, and HALT protease and phosphatase inhibitors, Thermo Scientific, Waltham, MA, USA) [25,26]. Following the addition of the lysis buffer, cells were scraped into 1.5 mL tubes and rotated at 4 °C for 30 min. Beads were isolated using a magnet and the supernatant was transferred to a new tube. Beads were washed three times in 500 μL of cold lysis buffer. Following the last wash, beads were resuspended in 40 μL cold PBS and 20 μL 3X SDS sample buffer. 

### 4.7. Generation of Anti-APC-M2 Chicken IgY Antibody

The central region of APC (amino acids 1001-1326, “APC-M2”) was cloned and ligated into the pET28b (Novagen Millipore Sigma, Burlington, MA, USA) expression vector, which contains an N-terminal 6X-His tag. Sequence-verified plasmids were transformed into BL21-CodonPlus-(DE3)-RIPL *E. coli* cells (NEB) for expression. Cells were grown in standard LB broth containing 50 μg/mL kanamycin at 37 °C with shaking (225 rpm) and induced with 0.2 mM isopropyl-β-D-thiogalactopyranoside (IPTG) for protein expression when an OD of 0.4–0.6 was reached. Cells were allowed to express induced protein products for 3–4 h at 37 °C with shaking before harvested by centrifugation at 4000 rpm, 15 min, 4 °C. Cellular pellets were resuspended in a buffer containing 50 mM Tris pH 8.0, 50 mM NaCl, 50 mM imidazole, 10% glycerol, and HALT protease cocktail (Thermo). Cells were lysed by a French pressure cell (35,000 psi), and the insoluble cellular debris was removed by centrifugation at 16,000× *g* for 45 min, 4 °C. Supernatant was applied to a chelating sepharose fast-flow column (Amersham Biosciences, Little Chalfont, UK) charged with nickel chloride and pre-equilibrated in resuspension buffer. Protein retained on the column was washed with a 3-column volume (C.V.) salt gradient (50 mM potassium phosphate pH 8.0, 500 mM NaCl, 50 mM imidazole, 10% glycerol). Protein was eluted with an imidazole buffer gradient (50 mM Tris pH 8.0, 500 mM NaCl, 500 mM imidazole, 10% glycerol). Fractions containing recombinant protein were pooled and applied to a Superdex 200 size-exclusion column (Amersham Biosciences) pre-equilibrated with 50 mM Tris, pH 8.0, 150 mM NaCl, and 10% glycerol, 1 mM DTT, and 1 mM octyl β-D-gluctopyranoside. Fractions containing recombinant protein were pooled and concentrated with Amicon Ultra centrifugal filters (Millipore). 

Recombinant APC antigen was shipped to Gallus Immunotech Inc. (now Exalpha Biologicals, Inc., Shirley, MA, USA) for injection into hens, and extraction of IgY-containing yolk extract. The anti-APC IgY was purified from the returned yolk extracts by affinity chromatography. Briefly, NHS-Sepharose (GE Healthcare, Chicago, IL, USA) was conjugated with APC-M2 antigen, purified as described above. 1M Tris (pH 8.0) and Tween-20 were added to the yolk extracts, to final concentrations of 10 mM and 0.2%, respectively. Buffered yolk extract was incubated with prepared APC-M2-conjugated NHS-Sepharose overnight at 4 °C. Columns were washed with multiple column volumes of PBS and PBS-T buffers. Anti-APC-M2 IgY was eluted from the column in 0.2 M glycine, pH 2.0. Fractions containing eluted anti-APC-M2 IgY were dialyzed into a buffer containing PBS at pH 7.4, 5% glycerol, and 0.01% sodium azide. 

### 4.8. Luciferase Reporter Assay

Cells were seeded into 12-well plates 24 h prior to transfection. On the day of transfection, cell culture media was changed one hour prior to siRNA and plasmid transfection. Both reporter plasmids and siRNAs were transfected concurrently using Lipofectamine 3000 according to the manufacturer’s instructions. Human APC-siRNA or control-siRNA was transfected as described above. For luciferase reporters, cells were cotransfected with TOPflash (450 ng) and Renilla (50 ng) expression plasmids. As a control, identical wells were transfected with FOPflash (450 ng) and Renilla (50 ng) expression plasmids to validate that a scrambled TCF-binding sequence does not result in increased reporter activity upon treatment. Following two days of APC-depletion, cells were treated with Wnt3a for 24 h and lysed using the Dual-Luciferase Reporter Assay System (Promega). Firefly luciferase signal was normalized to the Renilla luciferase signal, and data were normalized to control (set to 1). 

### 4.9. Immunofluorescence and Analysis 

Cells were briefly rinsed in PBS prior to fixation. Cells were fixed in 4% PFA in Brinkley’s Buffer 1980 (80 mM PIPES pH 6.8, 1 mM MgCl_2_, 1 mM EGTA) for 20 min at room temperature (RT) and washed two times in PBS prior to permeabilization in TBS + 0.2% Triton X-100 for 5 min. Cells were washed in TBS two times prior to incubation for 1 hour at RT in blocking buffer containing TBS + 0.2% Triton X-100, 1% BSA, and 3% Normal Goat Serum. Primary and secondary antibodies were incubated for 1 hour at RT. Cells were washed three times in TBS following primary and secondary antibody incubations. Mounting of coverslips was performed using Prolong Diamond Antifade Mountant with DAPI (Invitrogen). The anti-β-catenin mouse mAb (BD Transduction Laboratories, San Jose, CA, USA; #610154) was diluted 1:250 in blocking buffer. Stained cells were examined using an Axioplan microscope (Zeiss, Oberkochen, Germany) with a ×100 objective. Images of stained cells were captured using an Orca R^2^ digital camera (Hamamatsu, Hamamatsu City, Shizuoka, Japan). 

For calculation of the nuclear and cytoplasmic distribution of β-catenin, CellProfiler version 3.1.9 [57] was used to identify and measure the total and mean pixel intensities of β-catenin protein in cytoplasmic or nuclear compartments. Briefly, nuclei identification was first performed using the DAPI image, followed by cell identification by propagation. The cytoplasm was identified using the identified “total cells” and the identified nuclei to remove nuclei from the cytoplasmic calculation. Automated pipeline creation was performed using the following as a guide: [58]. 

### 4.10. Quantification and Statistical Analysis

Fluorescent-detection and quantification of immunoblots was performed using the LI-COR Odyssey CLx imaging system and Image Studio™ Lite software, version 5.2.5 (LI-COR, Lincoln, NE, USA). Graphs were generated using GraphPad Prism, version 8.4.3 (GraphPad Software, Inc.), and all statistical analyses were performed using a two-tailed, unpaired t-test in which a value of *p* < 0.05 is statistically significant. 

## 5. Conclusions

Using data available on cBioPortal, we find that mutations to β-catenin occur in ~6% of human CRC and in 9% of CRCs lacking an APC mutation. Combined with our previously published data demonstrating that the β-catenin destruction complex relocalizes toward a Wnt cue in cells expressing mutant β-catenin, we hypothesized that “stabilizing” β-catenin mutations do not completely evade Wnt regulation. It has been previously proposed that *APC* mutations occur in a “just-right” fashion to allow optimal levels of β-catenin signaling for tumorigenesis. The current work demonstrates that stabilizing β-catenin mutations allow cells to respond to Wnt and are prone to regulation by APC. Wnt treatment and APC-depletion both induce increased β-catΔS45 protein and activity by the TOPflash reporter assay. Interestingly, we find that Wnt treatment does not increase the nuclear accumulation of β-catΔS45 whereas APC-depletion results in predominantly nuclear β-catΔS45 localization. This data supports a role for APC in cytoplasmic retention of β-catenin. Further, we verify that β-catΔS45 can be phosphorylated at the GSK-3β phosphorylation sites, despite the lack of a priming Ser45. Treatment with proteasome inhibitors results in β-catΔS45 accumulation, indicating that this “stabilized” protein is still able to be targeted to the proteasome. Our results indicate that a Wnt signal leads to secondary effects that further activate nuclear β-catenin and that APC may inhibit this activity by nuclear export and/or sequestration of β-catenin in the cytoplasm. 

## Supplementary Materials

The following are available online at https://www.mdpi.com/2072-6694/12/8/2114/s1, Figure S1: Validation of anti-APC-M2 chicken IgY antibody, Figure S2: Full-length Western blots, Table S1: Mutations analyzed from cBioPortal including data from: MSKCC, TCGA, DFCI, and Genentech, Table S2: Liver Cancer Patient Samples with various CTNNB1 mutations queried for Wnt target gene RNA level.
