# Supplementary material for: Oncogenic Serine 45-Deleted β-Catenin Remains Susceptible to Wnt Stimulation and APC Regulation in Human Colonocytes
Source: Cancers (Basel). 2020 Jul 30;12(8):2114. doi: 10.3390/cancers12082114 (PMC7464804; doi:10.3390/cancers12082114)
Supplement: Supplementary file 1 [file cancers-12-02114-s001.zip › cancers-848197 supplementary.docx]

**Supplementary Materials:**

Oncogenic Serine 45-Deleted β-Catenin Remains Susceptible to Wnt Stimulation and APC Regulation in Human Colonocytes


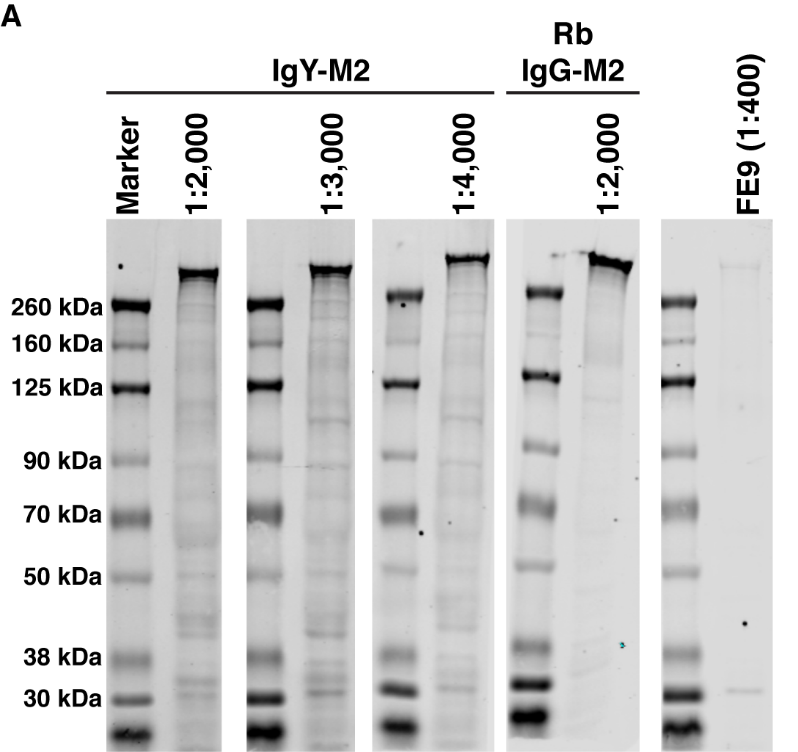


**Figure S1.** Validation of anti-APC-M2 chicken IgY antibody. Western blot of HCT116βm cells run on the same SDS-PAGE gel and transferred to 0.45μm nitrocellulose membrane. The membrane was cut into strips and probed with varying concentrations of the chicken IgY APC-M2 antibody, our previously developed rabbit APC-M2 antibody, or FE9 antibody. Each lane is run next to Chameleon Duo Ladder (LI-COR).


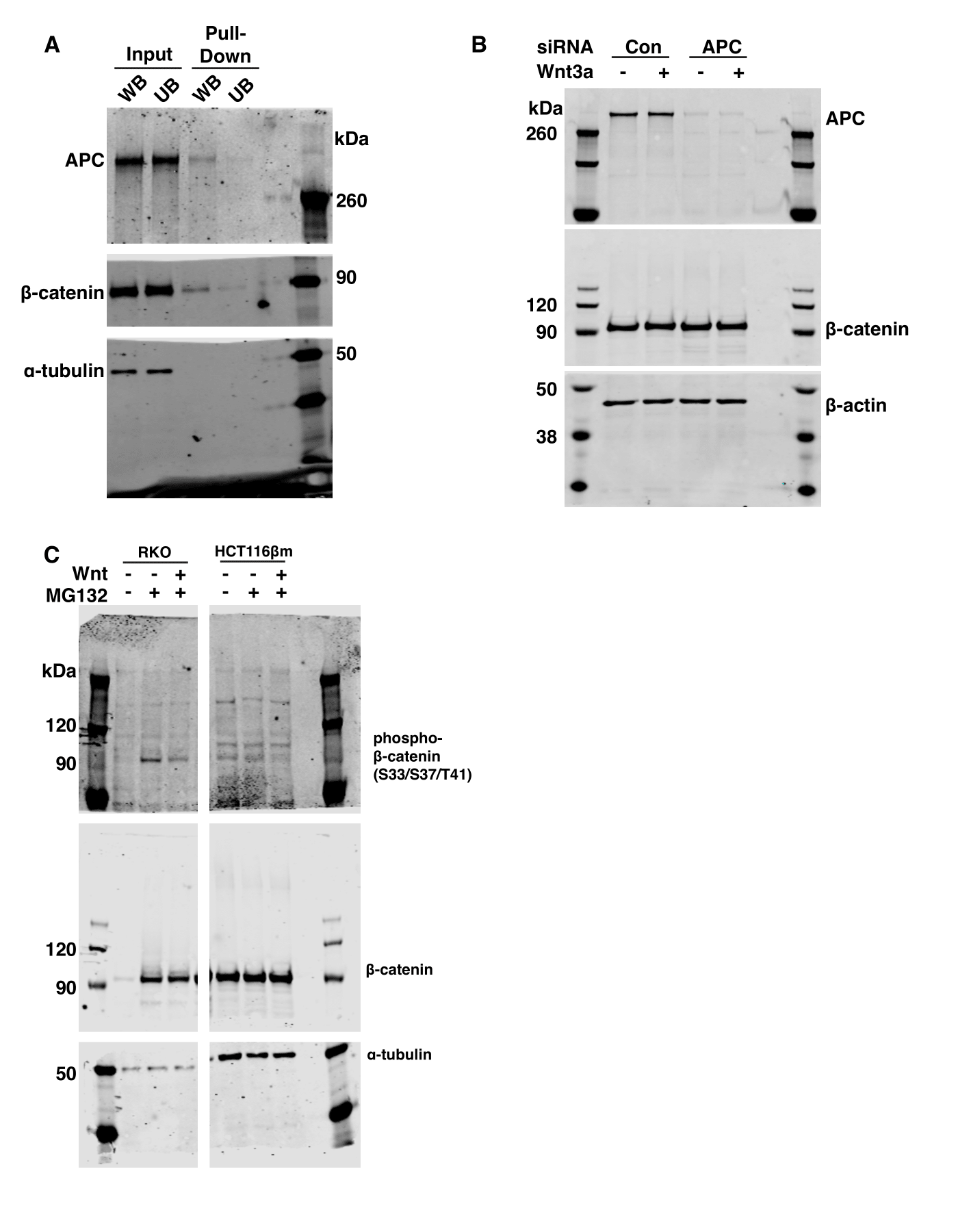


**Figure S2.** Full-length western blots.

**Table S1.** Mutations analyzed from cBioPortal including data from: MSKCC, TCGA, DFCI, and Genentech.

|  | **Mutation Frequency Among Patients** | | | |  |  |  |  |  |
| --- | --- | --- | --- | --- | --- | --- | --- | --- | --- |
|  | ***CTNNB1*** | ***APC*** | ***Axin1*** | ***Axin2*** |  |  |  |  |  |
| Patients Affected | 160 | 1620 | 70 | 150 |  |  |  |  |  |
| % Patients | 6.88% | 69.71% | 3.01% | 6.45% |  |  |  |  |  |
|  |  | | | |  |  |  |  |  |
| Total Patients | 2324 | Total Samples | | 2359 |  |  |  |  |  |
|  | **Number of Mutations for β-cat Destruction Complex Members** | | | |  |  |  |  |  |
|  | ***CTNNB1*** | ***APC*** | ***Axin1*** | ***Axin2*** |  |  |  |  |  |
| Missense | 131 | 223 | 56 | 68 |  |  |  |  |  |
| Truncating | 32 | 2324 | 23 | 85 |  |  |  |  |  |
| Inframe | 7 | 3 | 0 | 3 |  |  |  |  |  |
| Other | 6 | 8 | 0 | 0 |  |  |  |  |  |
| Total Mutations | 176 | 2558 | 79 | 156 |  |  |  |  |  |
|  |  |  |  | **Mutual Exclusivity** | | |  |  |  |
| A | B | Neither | A Not B | B Not A | Both | Log_2_ Odds Ratio | *p*-Value | *q*-Value | Tendency |
| *AXIN1* | *AXIN2* | 2158 | 48 | 129 | 22 | 2.939 | <0.001 | <0.001 | Co-occurrence |
| *CTNNB1* | *APC* | 632 | 86 | 1562 | 77 | −1.465 | <0.001 | <0.001 | Mutual exclusivity |
| *APC* | *AXIN2* | 652 | 1554 | 66 | 85 | −0.888 | <0.001 | <0.001 | Mutual exclusivity |
| *CTNNB1* | *AXIN1* | 2136 | 151 | 58 | 12 | 1.549 | 0.002 | 0.004 | Co-occurrence |
| *APC* | *AXIN1* | 688 | 1599 | 30 | 40 | −0.802 | 0.017 | 0.021 | Mutual exclusivity |
| *CTNNB1* | *AXIN2* | 2060 | 146 | 134 | 17 | 0.84 | 0.028 | 0.028 | Co-occurrence |

**Table S2.** Liver Cancer Patient Samples with various *CTNNB1* mutations queried for Wnt target gene RNA level.


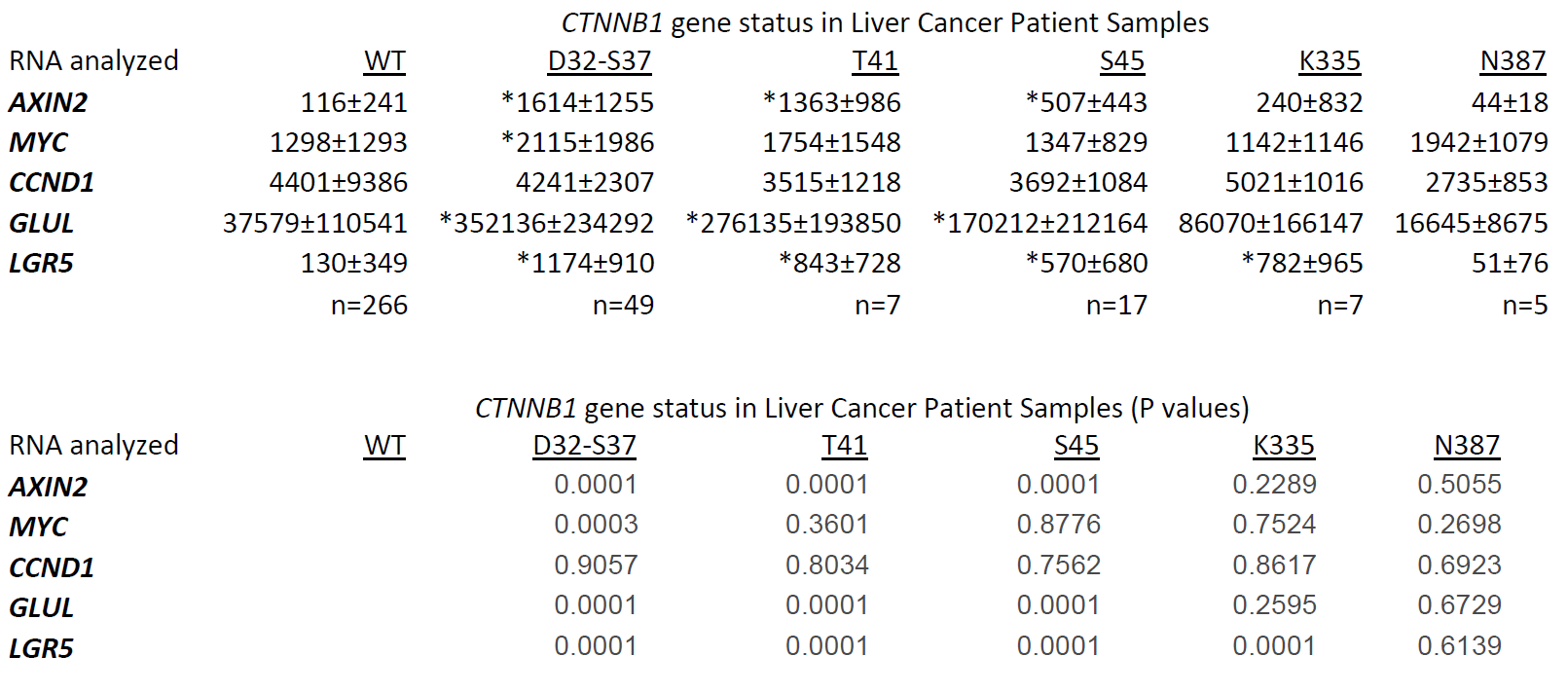


Values are presented as average with SD, * = *p* value < 0.05 by paired student *T* test when compared with WT samples.
